# An injectable hybrid nanoparticle-in-oil-in-water submicron emulsion for improved delivery of poorly soluble drugs

**DOI:** 10.1186/1556-276X-7-219

**Published:** 2012-04-13

**Authors:** Shuo Wang, Hua Wang, Wenquan Liang, Yongzhuo Huang

**Affiliations:** 1 Zhejiang University College of Pharmaceutical Sciences, 388 Yuhangtang Road, Hangzhou 310058, China; 2 Shanghai Institute of Materia Medica, Chinese Academy of Sciences, 501 Hai-ke Road, Shanghai 201203, China; 3 Key Lab of Smart Drug Delivery (Fudan University), MOE & PLA, 826 Zhangheng Rd, Shanghai 201203, China

**Keywords:** Poor solubility, Drug delivery, Nano emulsion, Nanoparticle, Dihydroartemisinin

## Abstract

Poor drugability problems are commonly seen in a class of chemical entities with poor solubility in water and oil, and moreover, physicochemical instability of these compounds poses extra challenges in design of dosage forms. Such problems contribute a significant high failure rate in new drug development. A hybrid nanoparicle-in-oil-in-water (N/O/W) submicron emulsion was proposed for improved delivery of poorly soluble and unstable drugs (e.g., dihydroartemisinin (DHA)). DHA is known for its potent antimalarial effect and antitumor activity. However, its insolubility and instability impose big challenges for formulations, and so far, no injectable dosage forms are clinically available yet. Therefore, an injectable DHA N/O/W system was developed. Unlike other widely-explored systems (e.g., liposomes, micelles, and emulsions), in which low drug load and only short-term storage are often found, the hybrid submicron emulsion possesses three-fold higher drug-loading capacity than the conventional O/W emulsion. Of note, it can be manufactured into a freeze-drying form and can render its storage up to 6 months even in room temperature. The in vivo studies demonstrated that the PK profiles were significantly improved, and this injectable system was effective in suppressing tumor growth. The strategy provides a useful solution to effective delivery of such a class of drugs.

## Background

Poor solubility of a chemical entity is a major obstacle affecting its drugability [1]. It is estimated that more than 40% of the new chemical entities generated in drug discovery programs are poorly soluble [2]. With growing interest in the role of modern pharmaceutics in early stage of drug discovery and development, rationale design of drug delivery systems for poorly soluble drugs has been widely explored, such as emulsions [3,4], solid lipid nanoparticles [5,6], micelles, and liposomes [7,8]. Emulsions are one of the most commonly used drug carriers for poorly water-soluble drugs due to the unique advantage in mass manufacturing, easy sterilization, and excellent physicochemical stability. However, there still remain two problems waiting to be solved, i.e., low drug-loading capacity and quick drug release. Moreover, for the drugs that remain insoluble in both water and oil, there is no satisfactory solution yet. As a case in point, dihydroartemisinin (DHA), a potent antimalarial drug [9,10], is poorly soluble and unstable, and its oral bioavailability is low due to degradation in the gastrointestinal tract and poor intestinal absorption. Moreover, because of its unfavorable solubility property, no injectable dosage form is clinically available yet, thus, severely limiting its use in medical emergency in severe malaria. According to WHO, an estimated 781,000 deaths from malaria in 2009, and most victims are children under five [11]. Furthermore, DHA recently has been demonstrated for its extraordinary antitumor activity and its promising development into anticancer drug [12-14]. Therefore, to develop an injectable drug delivery system for DHA is not only clinically beneficial for these diseases, but also such exploration is meaningful for rationale design of drug delivery for the class of water/oil insoluble drugs.

**Figure 1:**
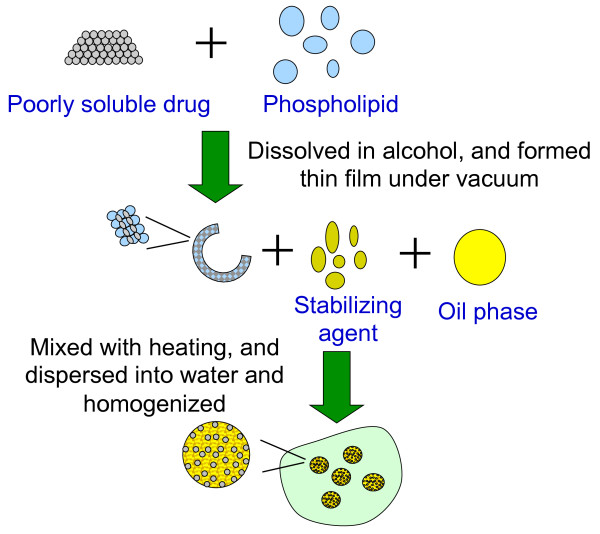
Schematic illustration of the preparation of nanoparticle-in-oil-in-water (N/O/W) submicron emulsion.

Herein, we propose a novel hybrid system of the nanoparticle-in-oil-in-water (N/O/W) submicron emulsion. Considering the oil compatibility, solid lipid nanoparticles (SLN) were selected. Drugs were incorporated into SLN and dispersed in oil as oily phase, and the oily phase was used to dispersed in aqueous solution with homogenization for preparation of the N/O/W submicron emulsion (Figure 1).

## Materials and methods

### Materials

Dihydroartemisinin (purity > 99%) was kindly provided by Beijing Zhongyan Tongrentang Medicine R&D Co., Ltd. (Beijing, China). Poloxamer 188 was purchased from BASF Corporation (Ludwigshafen, Germany), cholesterol from Pharmacia Biothech (Piscataway, NJ, USA), and soya phosphatidylcholine from Lipoid GmbH (Ludwighafen, Germany). Mannitol, sucrose, and glyceryl monostearate were purchased from Sinopharm Chemical Reagent Co., Ltd. (Shanghai, China). Hepatic H_22_ cell line was kindly provided by Zhejiang University of Traditional Chinese Medicine. All other chemicals were of analytical grade.

### Preparation of DHA N/O/W emulsion

DHA (150 mg) and lecithin (900 mg) were dissolved in 30-ml alcohol and then mixed with soybean oil (5,000 mg) and glyceryl monostearate (150 mg). The mixture was used as oily phase and warmed up to 60°C and then dropwise added to the aqueous phase containing glycerol (1,100 mg) and poloxamer 188 (900 mg) with agitation at high speed and temperature (8,000 to 10,000 rpm and 60°C). After adjusting the pH from 6 to 7, the primary emulsion was homogenized by microfluid technology at a pressure of 200 to 1,000 bar (8 to 10 cycles) to obtain the DHA N/O/W emulsion with DHA concentration about 3.0 mg/ml. In order to maintain DHA stability, the freeze-dried emulsion was prepared with a complex of sucrose and mannitol (4:1) as lyoprotectant.

Meanwhile, the DHA O/W emulsion was prepared as follows. DHA (49 mg) and lecithin (1,000 mg) were dissolved in alcohol and attached to a rotary evaporator to remove the organic solvent. Soybean oil (5,000 mg) was then added to dissolve the dried film as oil phase. Aqueous phase contained glycerol (1,100 mg); poloxamer, 188 (1,000 mg). The DHA O/W emulsion was prepared as described above.

### Characterization of the DHA N/O/W emulsion

Size and zeta potential of the DHA N/O/W emulsion were determined by laser scattering technique using Malvern ZS90 (Malvern Instruments Ltd., Malvern, Worcestershire, UK) after appropriate dilution with double distilled water.

The primary N/O/W emulsion with a size of about 10 μm (before microfluid process), through light microscopy (MPS-30, Leica Microsystems, Wetzlar, Hesse, Germany), was employed to observe the encapsulation of particles in O/W emulsion. Furthermore, morphology of the mid-product of DHA N/O/W emulsion (about 1 μm) was studied with transmission electron microscopy (TEM) (JEM-1200EX, JEOL Ltd., Akishima, Tokyo, Japan). The sample for TEM was dropped onto a 400-mesh copper grid coated with carbon film. The sample was dried in air before TEM observation. The sample for freeze-fracture transmission electron microscopy (FF-TEM) was prepared as follows. A droplet of microemulsion was sandwiched between two copper electron microscopy grids, snapfrozen by immersion in liquid ethane, and then loaded into a double-replica device and immersed in liquid nitrogen (−196°C). The double replica device was mounted on the rotary sample stage of a Balzers BAF-400D freeze etch device (Balzers AG, Balzers, Liechtenstein) cooled to −110°C. Fracturing was carried out at a vacuum of 3 × 10^−3^ mbar. The fractured surfaces were shadowed with platinum (45°) and carbon (90°). The replicas were then washed in chloroform, methanol, and finally, distilled water. Dried replicas were examined with TEM operating at an accelerating voltage of 80 kV.

### Drug loading capacity

The DHA N/O/W emulsion was dissolved in methanol and then injected to the high-performance liquid chromatography (HPLC) system. Chromatographic separation was achieved using a C_18_ column (150 × 4.6 mm, 5 μm, Agilent Technologies, Palo Alto, CA, USA) with column temperature at 40°C. The mobile phase consisted of 40:60 double-distilled water containing 0.1% acetic acid (Millipore Corporation, Milford, MA, USA)/acetonitrile (HPLC grade, Merck & Co., Inc., Whitehouse Station, NJ, USA). The flow rate was set to 1.0 ml/min, and UV detection wavelength was 210 nm.

### Pharmacokinetics study

The experimental protocols were approved by the Animal Ethics Review Committee of Zhejiang University. The male rabbits (2.0 ± 0.2 kg) were divided into three groups (6 per group). DHA solution, DHA O/W emulsion or DHA N/O/W emulsion was injected via ear vein at a dose of 6.2 mg/kg. Blood samples were collected from the marginal vein on another ear before administration and at predetermined time points after intravenous administration. Plasma sample (100 μl) was spiked with 10 μl of the IS solution (artemisinin, 1 μg/ml), and the mixture was vortexed for 10 s. The sample was extracted with 2-ml ethyl acetate, vortexed for 3 min, and centrifuged for 15 min at 3,500 rpm. The upper layer was transferred into another test tube and evaporated to dryness under a gentle stream of N_2_ gas at 37°C. The residue was reconstituted in 100 μl of methanol/water (60:40). The concentration of DHA in plasma samples was determined by LC-MS method (ESI, positive mode, Agilent 1100 LC-MSD, Agilent Technologies, Palo Alto, CA, USA). Pharmacokinetic analysis was conducted using Kinetica Software (Version 4.4, Thermo Electron Corporation, Waltham, MA, USA).

### In vivo antitumor efficacy of DHA N/O/W emulsion

Hepatic H_22_ tumor cells (5 to 6 × 10^6^ cells per mouse) were inoculated subcutaneously to the Institute for Cancer Research (ICR) mice (6 to 8 weeks, 22 to 26 g) at the right axilla. Tumors were measured by a vernier caliper, and its volume (*V*) was calculated as follows:

(1) $$V=d^{2}\times D/2,$$

where *d* and D are the shortest and the longest diameter of the tumor in mm, repectively.

When the tumor grew to a mean size of approximately 300 mm^3^, mice were treated intravenously (i.v.) with DHA solution, DHA O/W emulsion, or N/O/W emulsion.

### Hemolysis test and vascular irritation test

Blood sample was collected from New Zealand white rabbit (2.5 kg) ear vein. A 10-ml aliquot of defibrinated blood and 10 ml of saline were mixed and centrifuged. The supernatant was discarded, and the wash step was repeated three times until no red color was observed in the supernatant. Erythrocytes were diluted with saline to 2% (*v*/*v*). The DHA N/O/W emulsion at varying concentrations was mixed with 2% erythrocyte solution in equal volume. The morphology of the erythrocytes was observed under the microscope, and the color change of the solution was monitored with spectrophotometry (*λ* = 414 nm) for hemolysis evaluation. Hemolysis effect was calculated as follows:

(2) $$\text{Hemolysis}(\%)=\frac{\left(A_{\text{sample}}-A_{\text{negative}}\right)}{A_{\text{positive}}-A_{\text{negative}}}\times 100\%,$$

where *A*_sample_ is the absorbance of the tested sample; *A*_negative_, the absorbance of the negative control (i.e., mixture of equal volumes of 2% erythrocytes and saline); and *A*_positive_, the absorbance of the positive control (i.e., mixture of equal volumes of 2% erythrocytes and distilled water).

For vascular irritation test, DHA N/O/W emulsion with a DHA dose of 6.2 mg/kg was injected to the marginal ear vein once per day for 7 days. At the eighth day, the rabbits were killed by aortic bleeding under pentobarbital anesthesia. The vascular irritation was investigated by histological examination of the injection site.

### Statistical analysis

Each experiment was performed in triplicate, and the values were expressed as mean ± S.D. Statistical analysis was performed using Student's *t* test. Significant differences are demonstrated by symbols (*, **).

## Results and discussion

### Preparation and characterization of DHA N/O/W emulsion

DHA N/O/W emulsion was prepared by a modified solvent evaporation and membrane emulsification method. The drug-loading capacity was improved by the DHA N/O/W emulsion, in which DHA concentration was 3.0 mg/ml, over three times higher than that of the DHA O/W emulsion (0.98 mg/ml).

**Figure 2:**
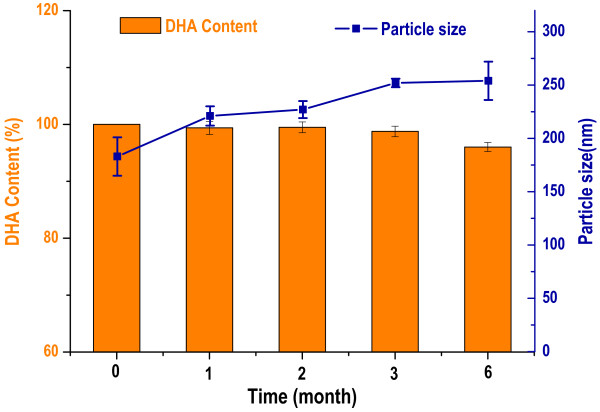
The changes of DHA content and size of the reconstituted N/O/W emulsion at different storage periods.

The freeze-dried DHA N/O/W emulsion was able to reconstitute in distilled water within 1 min. The average particle size and zeta potential were 175 nm and −48.1 mV. The storage stabilities of DHA N/O/W emulsion indicated that DHA N/O/W emulsion was stable at 4°C for 1 month. The stability was further improved in freeze-dry form, and the reconstituted emulsion showed merely slight changes in the average particle size and content of DHA at 25°C up to 6 months (Figure 2).

**Figure 3:**
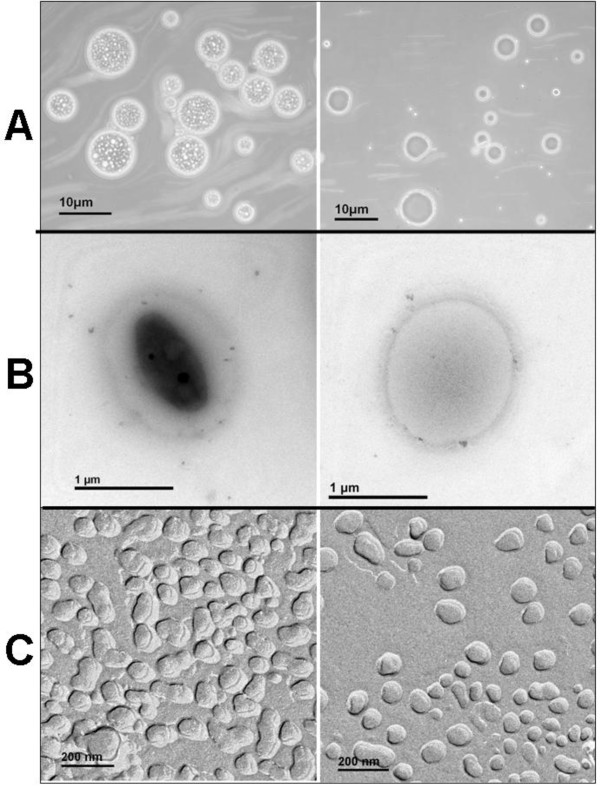
Light micrographs (A), TEM micrographs (B) and FF-TEM micrographs (C) of DHA N/O/W emulsion (left) and DHA O/W emulsion (right).

The representative light micrographs of the DHA N/O/W and DHA O/W emulsions are presented in Figure 3A. Particles were observed in the oil droplets of primary N/O/W emulsion, clearly showing the successful encapsulation of NP in oil droplets (magnification, ×400). As a control, no particles were seen in primary O/W emulsion. Such NP encapsulation in N/O/W emulsion (1 μm) was also found in TEM micrographs (Figure 3B). FF-TEM is a powerful tool to provide a direct image of the inside structures of nanomaterials and their morphology in a liquid sample, including emulsions [15] and nanoparticles [16]. Thus, FF-TEM was used to observe the internal structure of DHA N/O/W emulsion (Figure 3C). However, with the decrease in particle size (nanoscale), the difference between DHA N/O/W and DHA O/W emulsions becomes less apparent. It primarily contributed to the fact that the imaging resolution is not high enough to distinguish the internal cores (nanoparticulate core and nanodroplet oil core) under the freeze-fracture condition. Furthermore, the shell structure of the N/O/W becomes thinner with the decreasing particle size, and thus, the double layer of the shell is less apparent. However, a minor distinction can be seen that ‘wrinkles’ existed in the oil droplets of N/O/W submicron emulsion while not in O/W emulsion.

### Pharmacokinetics study

**Figure 4:**
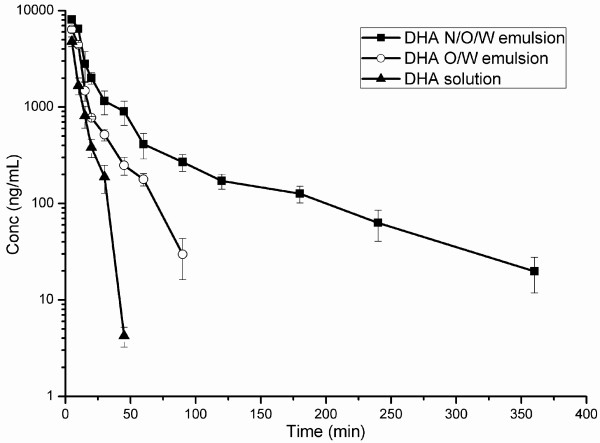
Plasma concentration-versus-time profiles of DHA after intravenous administration of 6.2 mg/kg DHA N/O/W emulsion to rabbits, DHA solution and DHA O/W emulsion as controls.

The plasma concentration-versus-time profiles are shown in Figure 4. The half-life of DHA was significantly prolonged in the group treated with DHA N/O/W emulsion compared with the group of the DHA O/W emulsion or the DHA solution. The pharmacokinetic parameters are listed in Table 1. In the animals treated with DHA solution, the blood DHA was rapidly cleared and hardly detected after 0.75 h, with a *t*_1/2β_ of 20 min. However, a very significant improvement was seen in the group of DHA N/O/W emulsion, showing eight-fold increase in *t*_1/2β_ (160 min). As a result, the area under the plasma concentration-versus-time curve (AUC) of DHA N/O/W emulsion group displayed nearly three times higher than that of the DHA solution group and 1.7 times higher than that of the DHA O/W emulsion. It demonstrated the advantages of improved PK profiles and the potential superior treatment outcomes of DHA N/O/W emulsion.

**Table 1:** Pharmacokinetic parameters of DHA

**Parameters**	**DHA N/O/W emulsion**	**DHA O/W emulsion**	**DHA solution**
*t*_1/2α_ (min)	9.40 ± 0.28*^,^**	3.97 ± 0.34	3.10 ± 0.09
*t*_1/2β_ (min)	160.91 ± 47.21*^,^**	73.31 ± 1.61*	20.20 ± 6.56
Cl (ml·min^−1^·kg)	0.0023 ± 0.0002*^,^**	0.0038 ± 0.0002*	0.0069 ± 0.0013
AUC (min·ng·ml^−1^)	219,759.00 ± 14,782.77*^,^**	131,564.5 ± 8,396.89*	73,025.35 ± 14,149.00
MRT (min)	97.65 ± 20.27*^,^**	32.22 ± 4.03*	9.40 ± 2.44

Parameters of DHA after intravenous administration of DHA N/O/W emulsion to rabbits, DHA solution, and DHA O/W emulsion as controls. The results are expressed as mean ± S.D. (n = 6), *P < 0.01 vs. DHA solution, **P < 0.01 vs. DHA O/W emulsion.

### In vivo antitumor efficacy of DHA N/O/W emulsion

**Figure 5:**
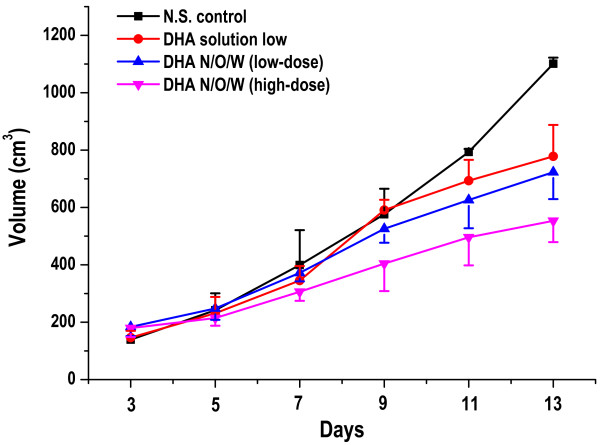
Mean relative tumor volume in DHA N/O/W emulsion (45 mg/kg), DHA N/O/W emulsion (11.25 mg/kg), DHA solution (11.25 mg/kg), and N.S. control. (n = 10).

Tumor-bearing mice transplanted with murine hepatic H_22_ cells were used to investigate the antitumor effect of DHA. During the treatment regime, tumor volume was monitored at a daily basis. Figure 5 shows the variation of tumor volume after H_22_ bearing mice were treated by i.v. injection of saline, DHA solution, and DHA N/O/W emulsion, respectively. The group treated with DHA N/O/W emulsion (45 mg/kg eq) displayed the strongest tumor growth inhibition and smallest tumor volumes (*P* < 0.01). In contrast, severe side effects (e.g., animal death) were seen in the group treated with DHA solution (45 mg/kg eq), indicating the over-tolerance dose. The tumor inhibition rate of DHA N/O/W emulsion was 51.8% (Table 2). Thus, it demonstrated the enhanced antitumor efficacy and the reduced toxicity of DHA with the N/O/W emulsion formulation.

**Table 2:** **In vivo anti-tumor effects of DHA and DHA N/O/W emulsion on growth of H**_**22**_**tumor in ICR mice (n = 10)**

**Group**	**Dose (mg/kg)**	**Body weight (g) (initial/endpoint)**	**Tumor weight (g)**	**TIR (%)**
NS control	-	21.9 ± 1.3/27.8 ± 2.2	1.4 ± 0.4	-
DHA solution	11.25	21.2 ± 1.2/25.3 ± 1.7	1.1 ± 0.3	21.5*
DHA N/O/W (low dose)	11.25	22.3 ± 0.9/28.6 ± 2.3	1.1 ± 0.2	23.0*
DHA N/O/W (high dose)	45.00	21.7 ± 1.1/26.3 ± 3.8	0.7 ± 0.2	51.8**

Note: ^*^P < 0.05 and ^**^P < 0.01 vs. N.S. control.

^#^*P* < 0.05 vs. DHA solution or low-dose group.

### Elementary safety assay

Hemolysis and vascular irritation evaluations are important for parenteral preparations before in vivo investigation because vessels and erythrocytes are the initial parts of the body that encounter and interact with i.v. injected preparations. Thus, the tests provide preliminary safety evaluation for DHA N/O/W emulsion. The DHA N/O/W emulsion displayed excellent biocompatibility. There were neither broken nor agglutinated erythrocytes observed under the microscope when exposed to the DHA N/O/W emulsion in the tested concentration range, and all samples showed less than 5% of hemolytic degree (Table 3).

**Table 3:** Hemolysis test of DHA N/O/W dry emulsion by spectrophotometry

	**Emulsion volume/total volume (%)**				**Negative control**	**Positive control**
	**13.3**	**10.0**	**6.7**	**3.3**		
Hemolytic degree (%)	3.2	1.5	1.6	1.5	0	100

**Figure 6:**
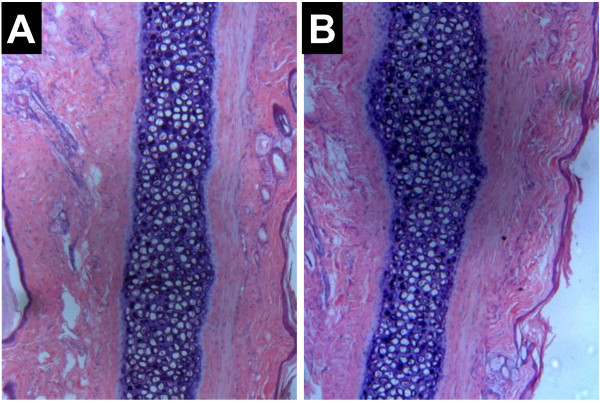
Histopathological sections of rabbit ears after intravenous administration of (A) DHA N/O/W emulsion and (B) Sodium chloride injection.

The vascular irritation test also demonstrated the safety of the DHA N/O/W emulsion in rabbits as shown in Figure 6. There was no swelling, thrombus, degeneration, or inflammatory cell infiltration observed in the histological examination after i.v. administration of the DHA N/O/W emulsion at the dose of 2.5 mg/kg. Our results indicated the biocompatibility of the injectable DHA N/O/W emulsion.

## Conclusion

A novel N/O/W submicron emulsion with high drug-loading capacity was prepared for intravenous administration of poorly soluble and unstable drugs. Our strategy is able to not only enhance the drug solubility in the emulsion but also improve its storage stability by freeze-drying. The pharmacokinetics of N/O/W emulsion are characterized by the significantly prolonged half-life and slow plasma clearance compared with conventional O/W emulsion and DHA solution. Presumably due to the improved PK profiles, the toxic effect of DHA was greatly reduced. Furthermore, the antitumor efficacy was demonstrated in vivo studies using a hepatic H_22_ tumor transplanted mouse model. More importantly, with the advantages of the high drug-loading capacity, easy manufacturing process, and excellent biocompatibility, the N/O/W emulsion provides a promising solution for developing injectable drug delivery systems for a class of drugs of poor water solubility and stability.

## Competing interests

The authors declare that they have no competing interests.

## Authors’ contributions

SW performed the experimental laboratory work. HW carried out the TEM and FF-TEM analyses. WL and YH conceived and designed the experiments. SW and YH prepared the manuscript. All authors read and approved the final manuscript.
